# A unique acromegalic osteoarthropathy: manubriosternal joint arthritis

**DOI:** 10.1002/ccr3.913

**Published:** 2017-03-29

**Authors:** Satoshi Fujisawa, Fumio Otsuka

**Affiliations:** ^1^ Endocrine Center of Okayama University HospitalOkayama University Graduate School of Medicine, Dentistry and Pharmaceutical Sciences2‐5‐1 Shikata‐cho, KitakuOkayama700‐8558Japan; ^2^ Department of General MedicineOkayama University Graduate School of Medicine, Dentistry and Pharmaceutical Sciences2‐5‐1 Shikata‐cho, KitakuOkayama700‐8558Japan

**Keywords:** Acromegaly, growth hormone, osteoarthropathy

## Abstract

Osteoarthritis of the manubriosternal joint can be found in patients with inflammatory arthritis. Acromegalic arthropathy is often found in the shoulders, wrists, knees, hips, and spine; however, attention should also be given to sternalgia as a complication of acromegalic involvement of the manubriosternal joint.

Question:

What do you think of chest pain as a complaint by acromegaly patients?

A 36‐year‐old male suffering from anterior chest pain was found to have increased levels of serum growth hormone (GH; 3.06 ng/mL) and insulin‐like growth factor‐I (IGF‐I; +5SD). A head X‐ray showed sellar expansion and acromegalic changes in the jawbone and frontal sinus (Fig. 1A). Cabergoline treatment was effective for normalization of serum IGF‐I levels for 6 years. As for his anterior chest pain with sternal tenderness, no sign of cardiovascular or pulmonary disorders was found. CT revealed irregularities of the joint spaces and osteosclerosis in the manubriosternal joint (Fig. 1B).

**Figure 1:**
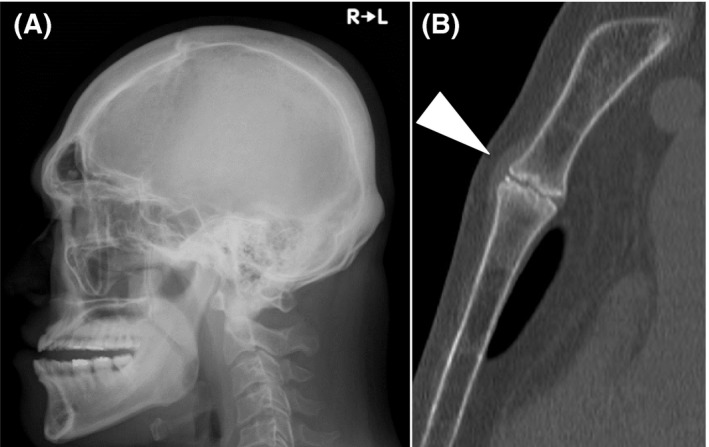
(A) Head X‐ray showed enlarged sinuses and an extended lower jaw. (B) Chest CT (sagittal section) showed irregularities of the joint spaces (white arrowhead).

Osteoarthritis of the manubriosternal joint can be found in patients with ankylosing spondylitis, rheumatoid arthritis, and SAPHO syndrome 1. Acromegaly patients often suffer from acromegalic arthropathy 2; however, attention should also be given to the possibility of sternalgia.

## Authorship

SF: wrote the manuscript and FO: who is the patient's doctor in charge, critically edited and revised the manuscript.

## Conflict of Interest

None declared.
